# Funktioneller Status und Lebensqualität von geriatrischen Patienten mit Wunden im Akutkrankenhaus: Ein Vergleich zwischen Patienten mit akuten und chronischen Wunden: eine Querschnittsstudie

**DOI:** 10.1007/s00391-021-01975-8

**Published:** 2021-10-05

**Authors:** Sylvie Lamotte, Anja Rappl, Ellen Freiberger, Cornel Christian Sieber, Thomas Johann Gehr

**Affiliations:** 1 Institut für Biomedizin des Alterns (IBA), Friedrich-Alexander-Universität Erlangen-Nürnberg (FAU), Nürnberg, Deutschland; 2 Institut für Medizininformatik, Biometrie und Epidemiologie (IMBE), Friedrich-Alexander-Universität Erlangen-Nürnberg (FAU), Erlangen, Deutschland; 3 Klinik für Innere Medizin, Kantonsspital Winterthur, Winterthur, Schweiz; 4 Stabstelle Pflegeentwicklung, Pflegedirektion, Krankenhaus Barmherzige Brüder Regensburg, Prüfeninger Straße 86, 93049 Regensburg, Deutschland

**Keywords:** Wundversorgung, Geriatrisches Assessment, Soziales Umfeld, Wohnsituation, Wound-Qol, Wound care, Geriatric assessment, Social environment, Living condition, Wound-Qol

## Abstract

**Hintergrund:**

Geriatrische Patienten sind alters- und krankheitsbedingt für das Auftreten einer Wunde, die den funktionellen Status und die Lebensqualität beeinträchtigt, prädisponiert. Dieser Aspekt wurde bisher in dieser Population wenig untersucht.

**Ziel:**

Das Ziel dieser Studie war es, den Einfluss von chronischer (cW) und akuter Wunde (aW) auf den objektiven funktionalen Status sowie auf die wundbezogene subjektive Lebensqualität bei hospitalisierten geriatrischen Patienten zu untersuchen.

**Methode:**

In dieser explorativen Querschnittsanalyse wurden die Daten von 41 Patienten mit Wunden untersucht, die in der Studie „Transsektorales Interventionsprogramm zur Verbesserung der Geriatrischen Versorgung in Regensburg“ (TIGER) (*n* = 244) rekrutiert wurden. Je nach Art der Wunde wurden die Patienten der Gruppe der aW (*n* = 19) oder cW (*n* = 22) zugeteilt. Die beiden Gruppen wurden hinsichtlich Mobilität, Handkraft, Alltagsaktivitäten, Emotion, Kognition und Ernährung sowie der wundbezogenen Lebensqualität (Wound-QoL) und soziodemografischen Daten miteinander verglichen.

**Ergebnisse:**

Zwischen der aW- und der cW-Gruppe ergab sich ein signifikanter Unterschied hinsichtlich des Geschlechtes (*p* = 0,045) und der Wohnsituation (*p* = 0,047). Die Art der Wunde war mit dem Barthel-Index (*p* = 0,010) und dem Wound-QoL (*p* = 0,022) assoziiert.

**Diskussion:**

Im Vergleich zu den aW-Patienten sind die cW-Patienten in den physischen und sozialen Dimensionen eingeschränkter und berichten von geringerer Lebensqualität. Die Wohnsituation von Alleinlebenden scheint dabei eine relevante Rolle zu spielen. Bei den Patienten der TIGER-Studie waren besonders alleinlebende Männer von cW betroffen. Die Betreuung dieser spezifischen Patientenpopulation sollte einen ganzheitlichen Ansatz verfolgen.

**Zusatzmaterial online:**

Die Online-Version dieses Beitrags (10.1007/s00391-021-01975-8) enthält eine kurze Beschreibung der durchgeführten Testungen und deren Literaturverweise.

Beitrag und Zusatzmaterial stehen Ihnen auf www.springermedizin.de zur Verfügung. Bitte geben Sie dort den Beitragstitel in die Suche ein; das Zusatzmaterial finden Sie beim Beitrag unter „Ergänzende Inhalte“.

## Abstract (en)

**Background:**

Geriatric patients are more predisposed to the occurrence of wounds due to age and disease, affecting functional status and quality of life. This aspect has rarely been researched in this population.

**Objective:**

The aim of this study was to investigate the influence of chronic (cW) and acute wounds (aW) on the objective functional status and wound-related subjective quality of life in hospitalized geriatric patients.

**Methods:**

In this exploratory cross-sectional analysis, data from 41 patients with wounds were examined. Patients were participating and recruited in the TIGER study (*n* = 244). Depending on the type of wound, patients were assigned to the aW (*n* = 19) or cW (*n* = 22) group. The two groups were compared in terms of physical function, hand strength, activities of daily living, depression, cognition, nutrition, quality of life (Wound-QoL) and sociodemographic data.

**Results:**

There was a significant difference between the aW and cW groups in terms of gender (*p* = 0.045) and living conditions (*p* = 0.047). The type of wound was associated with the Barthel index (*p* = 0.010) and the Wound-QoL (*p* = 0.022).

**Conclusion:**

Compared to aW patients, cW patients were more limited in the physical and social dimensions and reported a lower quality of live. Living alone seems to play a relevant role. Among the patients of the TIGER study, men living alone were particularly more affected by cW. The care for these specific patients population should follow a holistic approach.

## Hintergrund und Fragestellung

Die Wundbehandlung und -versorgung bei älteren Patienten stellt eine große Herausforderung dar, die eine biopsychosoziale Behandlung erfordert [4].

Eine Wunde ist definiert als ein „Barriereverlust zwischen dem Körper und der Umgebung durch Zerstörung von Gewebe an äußeren oder inneren Körperoberflächen“ [3]. Wundarten unterscheiden sich durch ihre Ätiologie und durch die Heilungsdauer. Eine akute Wunde (aW) entsteht durch äußere Einwirkungen und heilt im Rahmen der primären Wundheilung. Im Gegensatz dazu entwickelt sich die chronische Wunde (cW) durch eine zugrunde liegende Erkrankung und ist von den Wundheilungsphasen stark geprägt [16]. Sie wird als chronisch bezeichnet, wenn sie nach 8 Wochen keine Abheilung zeigt, wobei Wunden wie Ulcus cruris, diabetisches Fußsyndrom oder Dekubitus von Anfang an als cW gekennzeichnet sind [3]. Analog zu den cW beeinflussen lokale und systemische Faktoren (Infektion, Durchblutungsstörung, Diabetes mellitus) die Heilung einer aW [7], die bei älteren Patienten zur Wundheilungsstörungen führen kann [19]. Derzeit fehlt eine einheitliche internationale Definition für beide Ausprägungen der Wunde [22], wodurch die Spanne der Prävalenz und der Inzidenz in der Literatur stark variiert [12]. Da viele der über 80-jährigen Patienten eine alterstypische Vulnerabilität und Multimorbidität aufweisen [21], ist diese Altersgruppe für das Auftreten einer cW prädisponiert, was wiederum mit hohen Kosten einhergeht [6].

Die Wundbehandlung ist für viele Betroffenen mit einer Vielzahl an Einschränkungen verbunden. Das Spektrum umfasst sowohl die physischen, psychischen und sozialen Dimensionen des Individuums als auch finanzielle Belastungen [14]. Aus Studien über die negativen Auswirkungen von cW auf die gesundheitsbezogene Lebensqualität geht hervor, dass sie mit denen von Nieren- oder Herz-Kreislauf-Erkrankungen vergleichbar sind [6]. Im systematischen Review von Gorecki et al. [5] berichteten Menschen mit Druckgeschwüren über Ängste, Sorgen und Frustration.

Bereits 2009 plädierte Jaul [8] dafür, dass alterstypischen Faktoren wie Komorbiditäten, funktionellem Zustand, sozialer Unterstützung und Lebensqualität bei der Behandlung von Wunden älterer Menschen mehr Aufmerksamkeit gewidmet werden sollte. Es gibt aktuell kaum vergleichbare Studien, die über diese Einflüsse und Auswirkungen bei hospitalisierten geriatrischen Patienten sowohl mit cW als mit aW berichten. Gegenstand vieler Forschungsarbeiten sind die Wundbehandlung und -heilung primär von cW [6].

Das Hauptziel dieser Studie war, den Einfluss von cW und aW auf den funktionalen Status sowie auf die wundbezogene subjektive Lebensqualität von geriatrischen Patienten im Akutkrankenhaus zu untersuchen.

## Studiendesign und Untersuchungsmethoden

In der explorativen Querschnittsanalyse wurden die Daten von hospitalisierten geriatrischen Patienten mit aW und cW verglichen. Der Vergleich bezieht sich auf den funktionellen Status mit der Erhebung der Funktionalität, der Alltagsbewältigung, der Emotion, der Kognition, der Ernährung sowie der wundbezogenen subjektiven Lebensqualität.

### Studiendesign und Setting

Die verwendeten Daten wurden im Projekt „Transsektorales Interventionsprogramm zur Verbesserung der Geriatrischen Versorgung in Regensburg“ (TIGER) erhoben. Die randomisierte, kontrollierte Interventionsstudie wurde zwischen April 2018 und Juni 2020 in einem Akutkrankenhaus durchgeführt. Das Studienprotokoll wurde publiziert [18]. Ziel der Studie war, die Verbesserung der Versorgung geriatrischer Patienten an der stationär-ambulanten Schnittstelle zu untersuchen. Einschlusskriterien waren ein Mindestalter von 75 Jahren, eine Mitgliedschaft bei der AOK-Krankenversicherung, Rückkehr in die eigene Häuslichkeit sowie eine Mini-Mental State Examination (MMSE) von mindestens 22 Punkten und ein Wohnort im Einzugsgebiet von 50 km um das Krankenhaus [18]. Ausgeschlossen wurden Patienten in einer palliativen Versorgungssituation oder mit einer geplanten Wiederaufnahme innerhalb von 4 Wochen nach der Entlassung. Für diese Arbeit wurden diejenigen Patienten berücksichtigt, bei denen mindestens eine Wunde dokumentiert wurde. Je nach Art der Wunde wurden die Patienten in der Gruppe der aW oder cW zugeordnet. Ein positives Ethikvotum der Ethikkommission der Friedrich-Alexander-Universität Erlangen-Nürnberg sowie eine Einverständniserklärung von allen beteiligten Patienten liegen vor.

### Datenerfassung

Für die Beschreibung der Stichprobe wurden soziodemografische Daten wie Alter, Geschlecht, Familienstand, Bildung, Wohnsituation und Pflegegrad erhoben. Die Funktionalität wurde mit dem „Timed-up-and-go“(TUG)-Test und der Handkraftmessung erhoben, die Alltagsbewältigung mit dem Barthel-Index (ADL) und der Instrumental Activities of Daily Living Scale (IADL). Weiterhin wurden die Emotion mit der Geriatrischen Depressionsskala (GDS-15), die Kognition mit der MMSE und die Ernährung mit dem Mini Nutritional Assessment (MNA) erfasst. Zur Erhebung der wundbezogenen Lebensqualität bei Patienten mit cW wurde der valide, reliable und praktikable Wound-QoL-Fragebogen verwendet [1]. Er wurde auch für die aW-Gruppe verwendet, da kein vergleichbares Messinstrument für Patienten mit aW existiert. Die Schmerzintensität wurde mittels Selbstausfüllerfragebogen und die Anzahl der Medikamente aus dem Entlassungsbrief ermittelt. Die Erhebung der Daten erfolgte unmittelbar vor der Entlassung aus dem Krankenhaus nach dem Protokoll der TIGER-Studie [18]. Die verwendeten Instrumente sind im Supplement beschrieben.

### Datenanalyse

Die statistische Datenauswertung wurde mittels SPSS Statistics Version 26 (IBM, SPSS Inc., Chicago, IL, USA) durchgeführt. Für die Beschreibung der Stichproben und der Ergebnisse wurden die deskriptive Statistik und explorative Datenanalyse angewendet. Die Daten wurden gezielt nach Unterschieden hinsichtlich Alter, Geschlecht, Schmerzen, Anzahl der Medikamente, funktionellem Status und Lebensqualität untersucht. Die Daten wurden mittels relativer Häufigkeit (%), Mittelwert (MW), Standardabweichung (SD) und Median (M) mit unteren (Q1) und oberen Quartil (Q3) angegeben. Die Anzahl der Beobachtungen (*n*) wurde für jede Variable angegeben, woraus sich die fehlenden Daten ableiten lassen. Nominale Variablen wurden mittels Chi-Quadrat-Test verglichen, metrische Daten mit dem Mann-Whitney-U-Test. Das Signifikanzniveau wurde auf α = 0,05 festgelegt.

### Fehlende Daten

Die Erhebung einiger Dimensionen war wegen der Krankheitssituation des Patienten nicht möglich. Dies führte am häufigsten beim TUG-Test in der cW-Gruppe und bei der Handkraftmessung in der aW-Gruppe zu fehlenden Daten. Außerdem konnte der Gesamtscore des Wound-QoL nur berechnet werden, wenn mindestens 15 von 17 Items beantwortet wurden [1].

## Ergebnisse

Es wurden die Daten von 41 Patienten analysiert. Die soziodemografischen Daten, die alltagsrelevanten Parameter sowie die Wohnsituation der Patienten stellt Tab. 1 dar. Als Zusatzinformationen enthält Tab. 1 die Daten der TIGER-Patienten ohne Wunde. Die Ergebnisse der durchgeführten Assessments zeigt Tab. 2. Hinsichtlich Emotion, Kognition und Ernährung wurden keine Unterschiede zwischen beiden Gruppen festgestellt und deshalb nicht genauer betrachtet.

**Table 1:** 

Variable	Patienten ohne Wunde *n* = 203	Patienten mit aW *n* = 19	Patienten mit cW *n* = 22	Unterschied Patienten mit aW vs. cW *p*-Wert^a,b^
**Geschlecht** Männer, *n* (%) Frauen, *n* (%)	85 (41,8) 118 (58,2)	7 (36,8) 12 (63,2)	15 (68,2) 7 (31,8 %)	0,045*^,b^
**Alter **(MW ± SD)	82,04 ± 4,66	80,21 ± 4,36	82,59 ± 5,73	0,152^a^
**Schulbildung, *n*** ≤ 9 Jahre, *n* (%) ≥ 10 Jahre, *n* (%)	166 137 (82,5) 29 (17,5)	17 16 (94,1) 1 (5,9)	21 18 (85,7) 3 (14,3)	0,401^b^
**Wohnsituation/Unterstützung, *n*** Alleinlebend, *n* (%) Mit Partner/Angehörigen, *n* (%)	200 102 (51) 98 (49)	19 8 (42,1) 11 (57,9)	22 16 (72,7) 6 (27,3)	0,047*^,b^
**Pflegegrad, *n*** Kein Pflegegrad, *n* (%) Mit Pflegegrad, *n* (%)	175 124 (70,8) 51 (29,2)	18 12 (66,6) 6 (33,4)	19 7 (36,8) 12 (63,2)	0,070^b^

*aW* akute Wunde, *cW* chronische Wunde, *n* Anzahl der Fälle, *MW* Mittelwert, *±SD* Standardabweichung

^a^Mann-Whitney-U-Test

^b^Chi-Quadrat nach Pearson

*α < 0,05

**Table 2:** 

Variable	*n*	Akute Wunde Median (Q1;Q3)	*n*	Chronische Wunde Median (Q1;Q3)	*p*-Wert^a^
*Timed Up and Go-Test* (in Sekunden)	18	17,5 (9,0;26,0)	17	22,0 (18,5;27,5)	0,069
*Handkraftmessung* (in Kilogramm)	15	21,3 (18,0;32,6)	20	24,2 (17,2;32,6)	0,841
*Barthel-Index* (0–100)	19	95 (85;100)	22	85 (65;90)	0,010*
*Instrumentelle Aktivitäten des täglichen Lebens* (0–8)	19	7 (5,0;8,0)	22	5 (4,0;6,25)	0,060
*Mini-Mental State Examination* (0–30)	19	27 (27,0;28,0)	22	27 (26,0;28,0)	0,547
*Geriatrische Depressionsskala* (0–15)	19	2 (1,0;5,0)	22	3 (2,0;4,25)	0,551
*Mini Nutritional Assessment* (0–30)	19	24,0 (17,5;26,0)	21	21,5 (19,5;25,7)	0,914
*Wound-QoL* (0-4)	14	0,85 (0,47;1,73)	20	1,82 (1,33;2,47)	0,022*
*Schmerzen* (in den letzten 4 Wochen) (0–5)^b^	17	4 (1,5;4,5)	21	4 (3,0;4,0)	0,397
*Medikamente* (Anzahl)	17	8 (5,5;10,5)	20	10 (7,25;13)	0,073

*aW* akute Wunde, *cW* chronische Wunde, *n* Anzahl der Fälle, *Q1* 1. Quartil, *Q3* 3. Quartil

^a^Mann-Whitney-U-Test

^b^Skalenabfrage zwischen 0 (Nein, ich hatte keine Schmerzen) und 5 (Ja, ich hatte sehr starke Schmerzen)

*α < 0,05

### Deskription der Stichprobe

41 von 244 Patienten der TIGER-Studie hatten eine Wunde (16,8 %), 9 % eine cW (*n* = 22) und 7,8 % eine aW (*n* = 19). Die häufigste cW war das Ulcus cruris (*n* = 10, 4,1 %), gefolgt vom diabetischen Fußsyndrom (*n* = 8, 3,3 %) und als aW die Nahtwunde (*n* = 8, 3,3 %) und die Platzwunde (*n* = 6, 2,5 %). In der cW-Gruppe befanden sich mehrheitlich Männer, in der aW-Gruppe Frauen. Das Durchschnittsalter der aW-Patienten betrug 80,21 Jahre; die cW-Patienten waren um 2,38 Jahre älter. Die cW-Patienten lebten im Vergleich zur den aW-Patienten mehrheitlich allein (*p* = 0,047) und hatten zudem häufiger einen Pflegegrad (*p* = 0,070) (Tab. 1). Die TIGER-Patienten ohne Wunde waren mehrheitlich weiblich, hatten ein Durchschnittsalter von 82,04 Jahren, lebten zu in etwa gleichen Anteilen alleine oder mit Angehörigen und hatten überwiegend keinen Pflegegrad.

### Funktionalität

Die Funktionalität wurde durch die Mobilität (TUG-Test) und die Handkraftmessung erhoben (Tab. 2). Die zwei Gruppen unterschieden sich beim TUG-Test. Die cW-Patienten waren langsamer (M_cW_ (Q1–Q3): 22,0 (18,5–27,5) vs. M_aW_ (Q1–Q3): 17,5 (9,0–26,0), *p* = 0,069). Die Handkraft unterschied sich kaum zwischen den Gruppen (M_cW_ (Q1–Q3): 24,2 (17,2–32,6) vs. M_aW_ (Q1–Q3): 21,3 (18,0–32,6), *p* = 0,841).

### Alltagsbewältigung

Durchschnittlich hatten die cW-Patienten mehr Beeinträchtigungen im Bereich der Alltagsbewältigung sowohl beim Barthel-Index (M_cW_ (Q1–Q3): 85 (65–90) vs. M_aW_ (Q1–Q3): 95 (85–100), *p* = 0,010) als auch bei der IADL (M_cW_ (Q1–Q3): 5 (4,0–6,25) vs. M_aW_ (Q1–Q3): 7 (5,0–8,0), *p* = 0,060). Signifikant war der Unterschied beim Barthel-Index.

### Wundbezogene Lebensqualität

Hinsichtlich der Lebensqualität ergab die Befragung bei den cW-Patienten signifikant stärkere Beeinträchtigungen beim Wound-QoL (M_cW_ (Q1–Q3): 1,82 (1,33–2,47) vs. M_aW_ (Q1–Q3): 0,85 (0,47–1,73), *p* = 0,022).

### Gesundheitsbezogene Faktoren

Die cW-Patienten nahmen mehr Medikamente pro Tag als die aW-Patienten ein (M_cW_ (Q1–Q3): 10 (7,25–13) vs. M_aW_ (Q1–Q3): 8 (5,5–10,5); *p* = 0,073) (Tab. 2). Patienten mit aW gaben etwas weniger Schmerzen an (M_cW_ (Q1–Q3): 4 (3,0–4,0) vs. M_aW_ (Q1–Q3): 4 (1,5–4,5); *p* = 0,397). 75 % der cW-Patienten gaben an, mäßige bis sehr starke Schmerzen zu haben.

## Diskussion

Mit einer explorativen Analyse wurde der Einfluss von cW und aW auf den funktionellen Status und die wundbezogene subjektive Lebensqualität von hospitalisierten geriatrischen Patienten untersucht. Trotz großer Unterschiede in den Zahlen zu Prävalenz und Inzidenz von cW in der aktuellen Literatur [12], spiegelten unsere Ergebnisse (cW 9,0 %) vergleichbare Befunde der Studie von Caron-Mazet et al. [2] wider, die eine Prävalenz cW von 8,3 % (1163 Patienten in 14 geriatrischen Einrichtungen) fanden. In der Literatur fehlen Referenzwerte zu aW in geriatrischen Populationen. In unserer Studie war die Prävalenz aW 7,8 %. Die Gruppen unterschieden sich signifikant bezüglich Geschlecht und Wohnsituation. Alltagsbewältigung und Lebensqualität waren mit der Art der Wunde (aW vs. cW) assoziiert.

### Funktioneller Status

Beschriebene negative Auswirkungen cW auf ältere Menschen [8] konnten bezüglich Emotion, Kognition, Ernährung und Handkraft nicht repliziert werden. Beeinträchtigungen waren in den Bereichen Mobilität und Alltagsbewältigung feststellbar. Unterschiede in der Mobilität sind vermutlich v. a. durch die Lokalisation der cW am Bein (Ulcus cruris) oder am Fuß (diabetisches Fußsyndrom) erklärbar. Die cW-Patienten hatten erhöhte Defizite in der Selbstversorgung. In der Studie von Maresova et al. [11] wurde berichtet, dass Traumata, Wunden und Verletzungen zu Einschränkungen der Alltagsaktivitäten führen, und dass die Abnahme von Alltagskompetenzen durch mehr soziale Unterstützung reduziert werden kann. Diese Erkenntnisse könnten in der cW-Gruppe unserer Studie bedeuten, dass die Bewältigung der Alltagskompetenzen zusätzlich mit der Wohnsituation in Verbindung steht. Fast 60 % der Wundpatienten waren alleinlebend, davon hatten 66,7 % eine cW. Weitere Studien sollten untersuchen, ob eine Verbesserung der sozialen Situation sowohl einen Einfluss auf die Alltagskompetenzen als auch auf die Wundheilung haben kann.

### Lebensqualität

Die negativen Auswirkungen von cW auf die Lebensqualität ist in der Literatur mehrfach belegt [6]. Hier decken sich die Ergebnisse mit der aktuellen Studie von Reinboldt-Jockenhöfer et al. [17], die die Lebensqualität von 381 Patienten mit Ulcus multifaktorieller Genese aus wundspezifischen Zentren untersuchten. Sie fanden heraus, dass der Wound-QoL je nach Wundgenese zwischen 1,75 für das Ulcus cruris arteriosum und 1,92 für das diabetische Fußsyndrom variierte. Der Wound-QoL der cW-Gruppe ergab in unserer Studie einen Gesamtscore von 1,82. Der Gruppenvergleich zeigte, dass die Lebensqualität der cW-Patienten niedriger war als bei den aW-Patienten. Dies lässt sich neben dem Altersunterschied durch die retrospektive Erfassung der wundbezogenen Lebensqualität der letzten 7 Tage erklären. Es ist davon auszugehen, dass die Lebensqualität bei hospitalisierten Patienten, die vor der Krankenhauseinweisung an einer cW behandelt wurden, stärker durch die Wunde beeinflusst wird als bei Patienten mit einer aW. Jedoch fehlen Studien, die die Auswirkungen beider Wundarten im höheren Alter untersuchen.

### Geschlecht und Wohnsituation

In der cW-Gruppe waren mehrheitlich Männer, in der aW-Gruppe Frauen. Außerdem lebten die cW-Patienten eher allein. Laut der Studie von Khalil et al. [9] besteht kein Geschlechtsunterschied hinsichtlich der Prävalenz von aW und cW. Somit scheint weniger das Geschlecht als die Wohnsituation des Alleinlebens verantwortlich zu sein. Klein et al. [10] kommen in ihrer aktuellen Übersichtarbeit zu der Erkenntnis, dass soziale Unterstützung und Verbindungen bei Patienten mit cW reduziert sind. Familienmitglieder sind die wichtigsten sozialen Kontakte für Patienten, die sowohl Wundpflege als auch emotionale Unterstützung leisten [10]. Die negativen Auswirkungen sozialer Isolation und Einsamkeit auf Funktionalität, Lebensqualität und Gesundheit stellen gesundheitliche Risikofaktoren für älteren Menschen dar [15, 20].

## Limitationen

Da der Fokus der TIGER-Studie nicht spezifisch auf der Wundversorgung lag, gab es lediglich eine kleine Stichprobe an Wundpatienten. Die geringe Anzahl an Beobachtungen schränkte die statistischen Verfahren sowie Gruppenbildungen für weitere Analysemöglichkeiten ein. Da es sich um eine Querschnittanalyse handelte, kann nicht auf Kausalität geschlossen werden. Das Interesse, am TIGER-Projekt teilzunehmen, könnte bei alleinlebenden Männern mit cW höher gewesen sein. Um festzustellen, ob Wohnsituation und Geschlecht tatsächlich ein Risikofaktor bei Patienten mit cW sind, wären größere Stichproben notwendig. Eine weitere Limitation war die begrenzte Datenlage bezüglich geriatrischen Patienten mit Wunden.

## Ausblick

Im Vergleich zur aW-Gruppe war die cW-Gruppe stärker mit eingeschränkten Alltagskompetenzen und reduzierter Lebensqualität assoziiert. Die Auswirkungen der Wohnsituation auf die Wundheilung waren bisher nicht im Fokus der Forschung. Weitere Studien mit höherer Fallzahl sollten initiiert werden. Dies wäre für die klinische Praxis von großer Bedeutung und würde den Einsatz eines interdisziplinären transsektoralen Teams an der stationär-ambulanten Schnittstelle rechtfertigen [13]. Um festzustellen, welche Effekte die Wohnsituation auf die Wunde sowie auf die biopsychosozialen und ökonomischen Aspekte der Wundversorgung haben könnten, wären zudem Längsschnittstudien erforderlich.

## Fazit für die Praxis

- Bei geriatrischen Patienten mit chronischer Wunde sollten die Alltagsbewältigung und die Lebensqualität mitbeachtet werden.
- Wohnsituation und Geschlecht sollten als potenzielle Einflussfaktoren bei der Wundversorgung von geriatrischen Patienten miteinbezogen werden.
- Die Betreuung und Versorgung von Patienten mit chronischen Wunden bedarf einer ganzheitlichen Sichtweise.

## Supplementary Information




