# Supplementary material for: Functional status and quality of life of geriatric patients with wounds in acute hospitals: A comparison between patients with acute and chronic wounds: a cross-sectional study
Source: Z Gerontol Geriatr. 2021 Oct 5;55(1):32–7. [Article in German] doi: 10.1007/s00391-021-01975-8 (PMC8789624; doi:10.1007/s00391-021-01975-8)
Supplement: Supplementary file 1 [file 391_2021_1975_MOESM1_ESM.docx]

**Supplement: Kurze Beschreibung der durchgeführten Testungen und deren Literaturverweise**

| **Instrument/Anwendung** | **Kurze Beschreibung** | **Literaturverweis** |
| --- | --- | --- |
| Timed up and go-Test (TUG) | | |
| Test zur Messung der funktionalen Mobilität | Der Test misst die Zeit in Sekunden, die der Patient benötigt, um von einem Stuhl aufzustehen (mit Armlehnen; Sitzhöhe: ca. 46 cm), eine Strecke von 3 Metern zu gehen (mit oder ohne Hilfsmittel), sich umdrehen und zurück zum Stuhl zu gehen und sich dann wieder zu setzen. Es kann gegebenenfalls ein Hilfsmittel benutzen werden.  Interpretation der Ergebnisse:  ≤ 10 Sekunden: nicht eingeschränkt  11-19 Sekunden: leicht eingeschränkt  20-29 Sekunden: mäßig eingeschränkt  ≥ 30 Sekunden: stark eingeschränkt | Podsiadlo, D., Richardson, S. (1991) The Timed "Up & Go": A Test of Basic Functional Mobility for Frail Elderly Persons. Journal of the American Geriatrics Society:142-148. https://doi.org/10.1111/j.1532-5415.1991.tb01616.x. |
| Handkraftmessung | | |
| Test zur Messung der Handkraft | Der Test misst in Kilogramm die Handkraft mit dem JAMAR Hydraulic Hand Dynamometer. Der Patient sitzt bequem auf einen Stuhl und seine Unterarme liegen auf den Armlehnen. Nach der Anpassung des Messinstrumentes an der Handgröße der Patienten, wird er ermuntern so fest und so lange wie möglich zu drücken. Die Messungen werden abwechselnd wiederholt, um insgesamt drei Messungen für jede Seite zu erhalten. Der höchste gemessene Wert der sechs Griffkraftmessungen wird für die statistischen Analysen verwendet   \| Alter (Jahre) \|  \| Männer \| Frauen \| \| --- \| --- \| --- \| --- \| \|  \|  \| Kraft (Kg) Mean (95% CI) \| \| \| 75-79 \| Links \| 31.1 (25.6-36.6) \| 19.3 (16.1-22.4) \| \|  \| Rechts \| 33.0 (27.1-38.9) \| 21.6 (18.6-24.6) \| \| 80-84 \| Links \| 27.0 (22.2-31.8) \| 17.1 (14.5-19.6) \| \|  \| Rechts \| 30.1 (24.3-35.9) \| 17.3 (14.8-19.9) \| \| 85-89 \| Links \| 25.1 (20.5-29.7) \| 15.7 (12.2-19.2) \| \|  \| Rechts \| 25.8 (22.8-28.8) \| 17.1 (12.8-21.4) \| \| 90-99 \| Links \| 18.9 (17.4-20.3) \| 14.8 (11.2-18.4) \| \|  \| Rechts \| 18.8 (14.1-23.5) \| 15.2 (11.5-19.1) \| | Roberts HC, Denison HJ, Martin HJ et al. (2011) A review of the measurement of grip strength in clinical and epidemiological studies: towards a standardised approach. Age and ageing 40:423–429. https://doi.org/10.1093/ageing/afr051.  Bohannon RW, Bear-Lehman J, Desrosiers J et al. (2007) Average Grip Strength: A Meta-Analysis of Data Obtained with a Jamar Dynamometer from Individuals 75 Years or More of Age. Journal of geriatric physical therapy 30:28. |
| Barthel Index | | |
| Assessment zur Messung der Selbstversorgungsfähigkeiten in grundlegenden Alltagsaktivitäten und zur Abschätzung des Unterstützungsbedarfs | Die Fremdeinschätzung umfasst zehn alltagsrelevante Aktivitäten: Essen, Aufsetzen & Umsetzen, sich waschen, Toilettenbenutzung, Baden/Duschen, Aufstehen & gehen, Treppensteigen, An- & Auskleiden, Stuhlkontrolle, Harnkontrolle. Die Einstufungskriterien werden zwischen 0, 5, 10 oder 15 Punkten bewertet. Der Gesamtscore ist der Summenwert der 10 Items und beträgt zwischen 0-100 Punkte. Je geringer der Punktewert, desto pflegebedürftiger ist die Person.  Die Interpretation des Gesamtwertes:  ≤ 30 Punkte: weitgehend pflegeabhängig  35-80 Punkte: hilfsbedürftig  85-95 Punkte: punktuell hilfsbedürftig  100 Punkte: Zustand kompletter Selbständigkeit in den zugrunde liegenden Itembereichen und bezogen auf den jeweiligen | Lübke N, Meinck M, Renteln-Kruse W von (2004) Der Barthel-Index in der Geriatrie. Eine Kontextanalyse zum Hamburger Einstufungsmanual. Zeitschrift fur Gerontologie und Geriatrie 37:316–326. https://doi.org/10.1007/s00391-004-0233-2.  Mahoney F., Barthel D.W. (1965) Functional evaluation; the Barthel index. A simple index of the independence useful in scoring improvement in the rehabilitation of the chronically ill. Maryland State Medical Journal:61–65. |
| Instrumental Activities of Daily Living (iADL) | | |
| Assessment zur Beschreibung von Alltagsaktivitäten mit komplexen Anforderungen | Die Fremdeinschätzung umfasst acht Bereiche des täglichen Lebens, deren Bewältigung komplexe Anforderungen stellt: Telefon, Einkaufen, Kochen, Haushalt, Wäsche, Transportmittel, Medikamente und Geldhaushalt. Die Erhebung der Informationen erfolgt durch eine direkte Befragung, eine Befragung der Bezugspersonen mit Einbeziehung der Beobachtungen des Untersuchers. Die iADL-Items bilden eine achtstufige Skala für Frauen und eine fünfstufige Skala für Männer. Je geringer der Punktwert ist, desto abhängiger ist die Person bei der Durchführung von Alltagsaktivitäten mit komplexen Anforderungen. | Lawton MP, Brody EM (1969) Assessment of older people: self-maintaining and instrumental activities of daily living. Gerontologist 9:179–186. |
| Geriatrischen Depressions-Skala (GDS-15) | | |
| Assessment zur Erfassung der Emotion | Das Instrument besteht aus 15 Fragen, die zügig vorgelesen werden. Anschließend bittet der Untersucher der Patient spontan zu antworten. Der Patient kann nur mit „ja“ oder „nein“ beantworten.  Die Interpretation des Gesamtwertes:  ≤ 5 Punkte: unauffällig  6-10: leichte bis mäßiggradige Depression  ≥ 11 Punkte: Hinweise auf eine schwere Depression | Yesavage JA, Sheikh JI (1986) 9/Geriatric Depression Scale (GDS). Clinical Gerontologist 5:165–173. https://doi.org/10.1300/J018v05n01_09.  Gauggel S, Birkner B (1999) Validität und Reliabilität einer deutschen Version der Geriatrischen Depressionsskala (GDS). Zeitschrift für Klinische Psychologie und Psychotherapie 28:18–27. https://doi.org/10.1026//0084-5345.28.1.18. |
| Mini Mental State Examination (MMSE) | | |
| Assessmentinstrument zur Feststellung kognitiver Defizite | Das Instrument beinhalte 30 Items in Form von Fragen und umfasst folgende Bereiche: zeitliche und örtliche Orientierung, Arbeitsgedächtnis und Aufmerksamkeit, Neugedächtnis (Einspeicherfähigkeit, verzögerter Abruf), Benennen, Lesen und Schreiben sowie visuo-konstruktive Fertigkeiten.  Die Interpretation des Gesamtwertes:  ≤ 16: schwere kognitive Funktionseinschränkung  17-23 Punkte: mittlere kognitive Funktionseinschränkung  ≥ 24 Punkte: keine oder leichte kognitive Funktionseinschränkung | Folstein MF, Folstein SE, McHugh PR (1975) “Mini-mental state”. Journal of Psychiatric Research 12:189–198. https://doi.org/10.1016/0022-3956(75)90026-6. |
| Mini Nutritional Assessment Langform (MNA-LF) | | |
| Erfassung des Ernährungszustandes bei älteren Personen über 65 Jahren | Das Instrument ist in zwei Stufen aufgebaut: ein Screening mit 6 Fragen und ein Assessment mit 12 Fragen. Die Erhebung ist auf einer direkten Befragung, der Übernahme von Daten aus den Unterlagen und Messungen basiert. Maximal können 30 Punkte erreicht werden.  Die Interpretation des Gesamtwertes:  24-30 Punkte: normaler Ernährungszustand  17-23,5 Punkte: Risiko für Mangelernährung  ≤ 17 Punkte: Mangelernährung | Vellas B, Villars H, Abellan G et al. (2006) Overview of the MNA--Its history and challenges. The Journal of Nutrition Health and Aging 10:456-63; discussion 463-5. |
| Wound-Quality of Life (Wound-QoL) | | |
| Erfassung der krankheitsspezifischen gesundheitsbezogenen Lebensqualität bei Patienten mit chronischen Wunden | Der Fragebogen besteht aus 17 Items, mit denen die Beeinträchtigung der wundbezogenen Lebensqualität innerhalb der vergangenen sieben Tage erfasst wird. Er ist in drei Subskalen aufgebaut: Subskala „Körper“ (1-5), „Psyche“ (6-10) und „Alltagsleben“ (11-16). Der Item 17 wird keiner Subskala zugeordnet. Der Fragebogen wird selbstständig von den Patienten ausgefüllt. Der Patienten kann beim Ausfüllen eine Unterstützung erhalten. Der Gesamtwert wird auf einer 5-Punkte-Likert-Skala von 0 (gar nicht) bis 4 (sehr) bewertet. Dieser wurde nicht kalkuliert, wenn mehr als vier Items in den Subskalen nicht beantwortet wurden Je höher des Gesamtwertes, desto beeinträchtigter ist die wundbezogene Lebensqualität des Patienten. | Blome C, Baade K, Debus ES et al. (2014) The "Wound-QoL": a short questionnaire measuring quality of life in patients with chronic wounds based on three established disease-specific instruments. Wound repair and regeneration: official publication of the Wound Healing Society [and] the European Tissue Repair Society 22:504–514. https://doi.org/10.1111/wrr.12193.  Augustin M, Conde Montero E, Zander N et al. (2017) Validity and feasibility of the wound-QoL questionnaire on health-related quality of life in chronic wounds. Wound repair and regeneration: official publication of the Wound Healing Society [and] the European Tissue Repair Society 25:852–857. https://doi.org/10.1111/wrr.12583. |
| Schmerzintensität | | |
| Erfassung der Schmerzintensität | Analog zum deutschen Alterssurvey gibt diese Variable an, ob eine Person während der vergangenen 4 Wochen dauerhafte oder wiederkehrende Schmerzen hatte und wenn ja, wie stark diese waren. In einem Selbstausfüller-Fragebogen wurde die Schmerzintensität des Patienten erfasst.  Frage: „Hatten Sie während der vergangenen 4 Wochen dauerhafte oder wiederkehrende Schmerzen und wenn ja, wie stark waren diese?“ sollten Antwortmöglichkeiten:   - 0: Nein, ich hatte keine Schmerzen - 1: Ja, ich hatte sehr leichte Schmerzen - 2: Ja, ich hatte leichte Schmerzen - 3: Ja, ich hatte mäßige Schmerzen - 4: Ja, ich hatte starke Schmerzen - 5: Ja, ich hatte sehr starke Schmerzen | Klaus D, Engstler H, Mahne K et al. (2017) Cohort Profile: The German Ageing Survey (DEAS). International journal of epidemiology 46:1105-1105g. https://doi.org/10.1093/ije/dyw326. |
| Anzahl der Medikamente | | |
| Erfassung der Anzahl der Medikamente bei der Entlassung | Die Anzahl der unterschiedlichen Medikamente wurde aus dem Entlassungsbrief bei der Entlassung aus dem Krankenhaus erhoben. |  |
